# Presence of *Leishmania* and *Brucella* Species in the Golden Jackal *Canis aureus* in Serbia

**DOI:** 10.1155/2014/728516

**Published:** 2014-05-22

**Authors:** Duško Ćirović, Dimosthenis Chochlakis, Snežana Tomanović, Ratko Sukara, Aleksandra Penezić, Yannis Tselentis, Anna Psaroulaki

**Affiliations:** ^1^Faculty of Biology, University of Belgrade, Studentski Trg 16, 11000 Belgrade, Serbia; ^2^Regional Laboratory of Public Health of Crete, Heraklion, 71110 Crete, Greece; ^3^Laboratory for Medical Entomology, Department for Parasitology, Center of Excellence for Toxoplasmosis and Medical Entomology, Institute for Medical Research, University of Belgrade, 11000 Belgrade, Serbia; ^4^Laboratory of Clinical Bacteriology, Parasitology, Zoonoses and Geographical Medicine, Medical School, University of Crete, P.O. BOX 1393, Heraklion, 71110 Crete, Greece

## Abstract

The golden jackal *Canis aureus* occurs in south-eastern Europe, Asia, the Middle East, the Caucasus, and Africa. In Serbia, jackals neared extinction; however, during the last 30 years, the species started to spread quickly and to increase in number. Few studies in the past have revealed their potential role as carriers of zoonotic diseases. Animal samples were collected over a three-year period (01/2010–02/2013) from 12 sites all over Serbia. Of the tissue samples collected, spleen was chosen as the tissue to proceed; all samples were tested for *Leishmania* species and *Brucella* species by real-time PCR. Of the 216 samples collected, 15 (6.9%) were positive for *Leishmania* species, while four (1.9%) were positive for *B. canis*. The potential epidemiologic role of the golden jackal in carrying and dispersing zoonotic diseases in Serbia should be taken under consideration when applying surveillance monitoring schemes.

## 1. Introduction 

The golden jackal* Canis aureus* occurs in south-eastern Europe, Asia, the Middle East, the Caucasus, and Africa [1, 2]. During 20th century European part of its distribution was dynamic. At the beginning of the century, the population of the golden jackal in Europe declined dramatically; however, it has recently achieved to recolonization of its former territories [2] establishing local populations with high densities [3]. Currently European populations are both still rapidly increasing and widening their ranges toward Central and Eastern Europe [2, 4] making it one of the most numerous carnivore species.

In Serbia, jackals neared extinction due to extensive poisonings organized after World War II, initially aimed at controlling the size of the wolf population and lessening the damage they caused to domestic animals. Only two relic populations survived in Srem and in eastern Serbia near Bulgarian borders [5, 6]. At the beginning of the 1980s, the species started to spread quickly along the rivers (Timok, Morava, Danube, and Sava) and to increase in number [7, 8], which resulted in the fusion of the two relic populations and the widening of their range [9]. This range now covers more than half of the Serbian territory [10] with local populations that have the highest density at the Balkan Peninsula [3].

The golden jackal is described as an opportunistic species, capable of using a wide range of food sources [11]. Studies conducted in the Balkan region shows the predominance of anthropogenic food sources during the winter period [12–14]. This predominance leads to the positive relationship between jackals and human settlements. In Greece jackal groups were recorded at the average distance of only 2.61 km from the closest settlement [15, 16].

Increasing densities and widening of its European populations have not been accompanied by research of the presence of pathogens of interest both to animals and to humans. Only few studies have evaluated the potential of jackals as carriers of zoonotic diseases in Europe, while none has been conducted so far in the Serbian territory.

The scope of the current survey was to study the presence of two zoonotic agents,* Brucella* species and* Leishmania* species in the jackal population in Serbia and to evaluate its importance for animals and human health.

## 2. Materials and Methods

### 2.1. Animal Sampling

**Figure 1:**
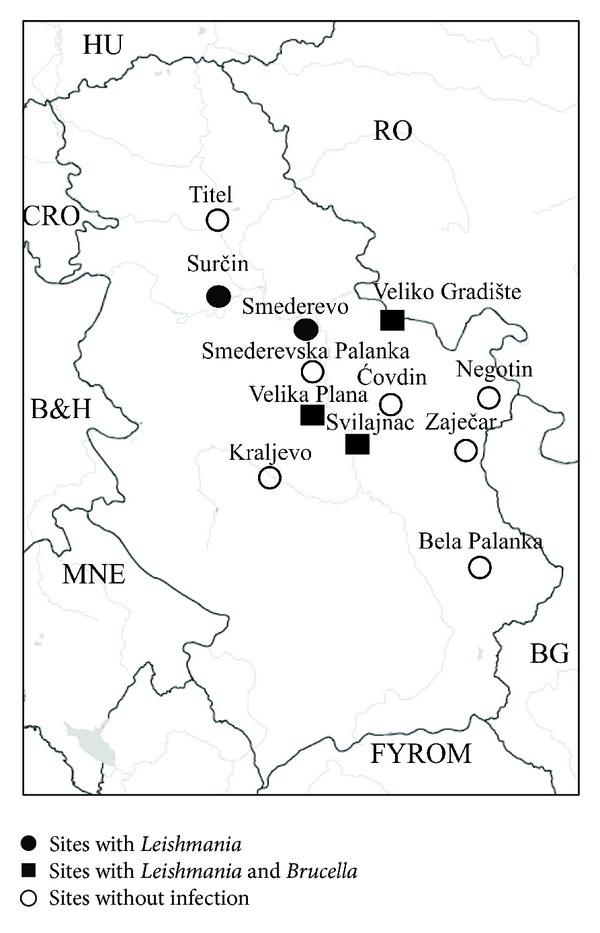
The sites where golden jackals were collected from and the corresponding results after testing for* Leishmania* and* Brucella* species by PCR.

Animal samples were collected over a three-year period (01/2010–02/2013) from 12 sites (Smederevo, Surčin, Veliko Gradište, Velika Plana, Svijalnac, Zaječar, Bela Palanka, Titel, Ćovdin, Smederevska Palanka, Kraljevo, and Negotin) all over the country (Figure 1), in cooperation with local hunting organizations.

Sampling took place from dead animals brought at the Laboratory for animal ecology (Faculty of Biology, Belgrade-Serbia) by hunters. Data regarding location, sex, and date (of death) of each jackal were recorded. At necropsy, samples from different tissues were collected (spleen, liver, lung, heart, and kidneys) and stored at −80°C until further processing.

### 2.2. Sample Processing

Of the tissue samples collected, spleen was chosen as the tissue to proceed with the testing, based on the increased possibility, among the tissues collected, to detect the pathogens of interest. Tissue samples were processed by homogenizing frozen pieces of spleen using micropastles. A small portion of smashed tissue was then removed and processed using the Gene Jet Genomic DNA Purification Kit (Fermentas, Thermo Scientific). All DNA extracts were stored at −20°C until PCR analysis.

### 2.3. Detection of* Brucella* and* Leishmania* Species by Real-Time PCR

Real-time PCR assays were carried at the Laboratory of Clinical Bacteriology, Parasitology, Zoonoses and Geographical Medicine of the University of Crete (Greece), where part of the DNA extracts was brought and always stored at −20°C. DNA extracts were initially pooled, at pools of five, at a final volume of 20 *μ*L. In case a positive PCR was detected, we went back to each pool and tested each corresponding DNA extract seperately.

For the detection of* Brucella *species, a multiplex real-time PCR protocol was used, targeting the* bcsp31*,* alkB,* and* BMEI1162* genes of Brucella species,* B. abortus, *and* B. melitensis*, respectively, as previously described [17]. Where it was positive for Brucella species but negative for the other two,* B. canis *was tested using a PCR protocol targeting the* omp2B* gene as previously described [18].

For the detection of* Leishmania* species a real-time PCR protocol targeting the SSU rRNA gene was used, as previously described [19].

All real-time PCR assays were carried out on a C1000 Touch, CFX96 thermal cycler (Biorad). At a final volume of 20 *μ*L, 10 *μ*L of SsoFast Probes Supermix was used as a mastermix together with 0.6 *μ*L (in the case of* Brucella* species) or 1.6 *μ*L (in the case of* Leishmania *species) of each primer (10 *μ*M), 0.4 *μ*L (in the case of* Brucella* species) or 0.2 *μ*L (in the case of* Leishmania *species) of each of the probes (10 *μ*M), 2.7 *μ*L (in the case of* Brucella* species) or 4.1 *μ*L (in the case of* Leishmania *species) of sterile water, and 2.5 *μ*L of the corresponding DNA.* Brucella melitensis* and* Leishmania infantum* isolated from human patients were used as positive controls for the corresponding assays. Two sets of negative controls (DNA from noninfected specimens and sterile water) were applied.

### 2.4. Statistical Analysis

Chi-square (SPSS v. 19) was applied to compare results in-between gender and among sampling years. In all cases a* P* value < 0.05 was considered as significant.

## 3. Results

A total of 216 spleen samples, collected from 48 localities corresponding to 12 sites of Serbia (Figure 1), were tested. Most samples (196/90.7%) were collected during the hunting period (November–February). Of the samples tested, 108 animals were male and 108 were female (Table 1).

Of the 216 samples, 15 (6.9%) (collected from Smederevo, Surčin, Svilajnac, Velika Plana, and Veliko Gradište) were positive for* Leishmania* species, while four (1.9%) (collected from Svilajnac, Velika Plana and Veliko Gradište) were positive for* B. canis*. A single pool of those initially tested was also positive for* Brucella* species; however, when we went back to test each corresponding DNA extract separately we could not trace which was the positive one, perhaps due to very low initial DNA concentration. In that single case, we went back to the original tissues, collected multiple samples from each tissue, and performed individual PCR amplifications always, however, getting negative results. None of the samples which tested positive for* Brucella* species was positive for* B. abortus *or* B. melitensis*.

As expected, based on the distribution of the collected samples throughout the year, all positive samples were recorded during the winter (hunting period) both for* Leishmania* species and for* B. canis*. Although the number of samples did not fluctuate dramatically from 2011 to 2013, a remarkable increase in the number of positive samples recorded for* Leishmania* species (*P* = 0.011), but not for* B. canis* (*P* = 0.436), was recorded during 2013. As regards the distribution over the various regions, it was noted that the higher the number of samples collected from a certain region, the higher the possibility to retrieve a positive sample. Concerning gender,* Leishmania* species showed a preference to males over females (*P* = 0.061), contrary to the results for* B. canis* (*P* = 0.313).

All results are summarized in detail in Table 1.

## 4. Discussion

Wildlife is considered as playing a crucial role in the maintenance and dispersal of leishmanioses and reservoirs may include rodents, marsupials, edentates, monkeys, domestic dogs, and wild canids [20].

Jackals are known to be omnivores and scavengers and they usually prey on small mammals but on vegetables, fruit, and garbage as well. Their ability to adapt to novel habitats both on rural and urban areas allows them to come into contact with animals that live in close proximity to humans, such as dogs, hence their ability to host a number of zoonotic pathogens, such as canine viruses [21],* Ehrlichia canis *[22],* Leishmania donovani* [23, 24], and* Echinococcus granulosus *[25], as well as ectoparasites like ticks and fleas in areas where they occur at high densities, which may raise this species as a crucial key in the chain of zoonotic diseases maintenance and dispersal.

According to ECCMID, visceral leishmaniasis is endemic, amongst other countries, in Serbia as well [26]. In fact the disease had been characterized as endemic in southern Serbia even when Yugoslavia still existed. During the 90s, 39 cases of leishmaniasis were recorded in Serbia and Montenegro, with the incidence rate in the range of 0.01/100.000 [27]. Nevertheless, the animal reservoirs are still unknown.

In a study recently conducted in Hungary in sera collected from jackals, no evidence for the presence of leishmaniasis was noted [28]. To our knowledge no other study has been published in regards to the study of* Leishmania* species in jackals.

Away from the European territory, there have been reports that described the presence in jackals of these agents, such as visceral leishmaniasis [29] in Israel and of* L. infantum* in Algeria [30]. In fact, in Israel, the reappearance at the mid 90s of human infections of visceral leishmaniasis in areas where it normally did not exist, pointed towards the study of* L. infantum* in wildlife (wild canids, jackals, and red foxes), recording a seroprevalence of 7.6%, which, taking into account the presence of the pathogen in dogs from the same regions, led the public authorities to raise suspicions on the possible role of wildlife in spreading this disease [24]. In a later seroepidemiological study conducted in 2001 in Israel in samples collected from healthy adult free-ranging golden jackals, a prevalence raising up to 6.5% was recorded [31], while in a more recent study conducted in 2010 [32], a prevalence of 7.8% was recorded with* L. tropica* being added to the species of* Leishmania* that jackals can host; results of both studies resembled the ones we came up with (6.9%).

Getting further away from Europe, the presence of* L. infantum* has been reported in golden jackals in Iraq [33] and in Kazakhstan [34], while in Iran, where sporadic cases of visceral leishmaniasis are reported from time to time, the role of jackals in the dispersal of the pathogen has been documented, with prevalence rising up to 12.5% [35–37].

As far as Brucellosis is concerned, Serbia is considered as a country with relatively low numbers of incidence rate (0.15/100.000) amongst the rest of Balkan countries and with a relatively low incidence rate within the European Union [38]. Nevertheless, more than 1500 cases of human Brucellosis have been recorded during the past 30 years, most of them occurring at the areas of Vojvodina and the southern part of central Serbia. Migration of animals among countries has been considered as the main reason of the spreading of old and the establishment of new foci of the pathogen [39].

In Europe,* Brucella *species have been identified in wild boar in France and Italy, in brown hare in Austria, France, the Czech Republic, and Switzerland, and in chamois and red deer in the Alps. Recently, its presence was also recorded in free-roaming domestic pigs, an indication that perhaps a wild reservoir may act as a source of the agent [40]. As regards the golden jackal, little, if any, work has been done on ability of this species to carry the pathogen. In fact only in Africa has the role of jackals been investigated superficially, on the dispersal of the pathogen. In a work done in Namibia studying the seroprevalence of* Brucella *in sheep and springbok [41] it was suggested that little evidence of abortions was recorded in farms that were recorded with high prevalence of* Brucella*, and this observation was attributed to the role of jackals and other carnivores in removing aborted fetuses and feeding on fetal membranes. During the late 60s, some evidence was recorded on the presence of* Brucella* species in jackals, in a study conducted in Tanzania [42]. The relatively low prevalence (1.9%) recorded in our study may suggest that perhaps jackals may not act as a primary reservoir of* Brucella* species; nevertheless, their tendency to scavenge carcasses and perhaps share areas of grazing with animals of veterinary importance may not exclude them as a potential source of dispersal of the pathogen.

An increase in jackal population has lately been observed in Serbia [7], while complaints about damages that jackals may cause to livestock mean that its wildlife cycle may well interfere with areas of natural human activity [43]. Their habitats are in proximity to densely populated areas while they are phylogenetically closely related to dogs. These facts, combined with the high prevalence of the jackals' exposure to the major canine pathogens demonstrated in this study, suggest that they may serve as a reservoir for the transmission of certain diseases to domestic dogs. The tendency nowadays is to use modeling and predictive risk maps in an attempt to describe areas that based on factors such as animal population density, climate, land surface, altitude, and so forth may prove in the future of being at high risk due to spread of the agents of interest. In the case of leishmaniasis, such an attempt has already been made to describe incidence maps and prediction maps using published data of seroprevalence studies on canine leishmaniasis [44]. The model worked well when focusing on a single country; however, since that was the first attempt to approach epidemiology through a more holistic perspective, much can be improved in order to be able to achieve better results. Similar approaches have been tried with respect to the population of sandflies. Studies performed in Central Europe have shown that increase in temperature may lead to an expansion of the population of sandflies leading, as a consequence, to an increase of the occurrence of sandfly-borne diseases such as leishmaniasis [45]. This, together with the introduction of infected dogs from nearby countries, could result in the establishment of the pathogen in areas which were otherwise free, together with the appearance of autochthonous cases. Other groups which work in countries in close proximity to Serbia have performed studies on leishmaniasis on other chain links of the wildlife, such as wolves [46], red foxes [47], or vectors through entomological studies on areas of high incidence of the disease [48], in an attempt to reveal new reservoirs of the pathogen or new foci that could give burst to human cases.

In any case, further study is required to discern the potential epidemiologic role of the golden jackal in spreading and transmitting the above studied pathogens not only within Serbia but to neighbor countries as well; local awareness of residents, veterinarians, and health professionals would prove of great importance in order to efficiently monitor these and other zoonotic diseases.

## 5. Conclusion

The golden jackal is a carrier of a number of zoonotic diseases as has been recorded in the past. Herein we described the potential role of the golden jackal as a carrier of* Leishmania* and* Brucella* species in Serbia. Its growing population and its capacity to spread and interfere with regions of animals of veterinary importance and/or of humans should point out a hint on future surveillance schemes on these two pathogens.

## Figures and Tables

**Table 1:** Distribution of samples collected from jackals and prevalence of positivity, recorded by PCR means, based on month, year, region, and gender.

	Samples collected (number)	Samples positive for *Brucella canis* (number/%)	Samples positive for *Leishmania* species (number/%)
Month			
Jan	64	1 (1.6)	6 (9.4)
Feb	75	2 (2.7)	4 (5.3)
Mar	6	0	0
Apr	2	0	0
May	6	0	0
Jun	1	0	0
Jul	3	0	0
Aug	2	0	0
Sept	0	0	0
Oct	0	0	0
Nov	10	0	0
Dec	47	1 (2.1)	5 (10.6)
Year			
2010	24	1 (4.2)	2 (8.3)
2011	80	0	1 (1.3)
2012	45	1 (2.2)	2 (4.4)
2013	67	2 (3.0)	10 (14.9)
Region			
Bela Palanka	4	0	0
Ćovdin	1	0	0
Kraljevo	3	0	0
Smederevo	49	0	6 (12.2)
Smederevska Palanka	3	0	0
Srbovo	4	0	0
Surčin	24	0	2 (8.3)
Svilajnac	44	1 (2.3)	4 (9.1)
Titel	5	0	0
Velika Plana	25	1 (4)	1 (4)
Veliko Gradište	54	2 (3.7)	2 (3.7)
Zaječar	2	0	0
Gender			
Male	108	1 (0.9)	11 (10.2)
Female	108	3 (2.8)	4 (3.7)

Total	216	4 (1.9)	15 (6.9)
